# RNA-seq analysis reveals considerable genetic diversity and provides genetic markers saturating all chromosomes in the diploid wild wheat relative *Aegilops umbellulata*

**DOI:** 10.1186/s12870-018-1498-8

**Published:** 2018-11-08

**Authors:** Moeko Okada, Kentaro Yoshida, Ryo Nishijima, Asami Michikawa, Yuka Motoi, Kazuhiro Sato, Shigeo Takumi

**Affiliations:** 1 Graduate School of Agricultural Science, Kobe University, Rokkodai 1-1, Nada-ku, Kobe, 657-8501 Japan; 2 Institute of Plant Science and Resources, Okayama University, Kurashiki, Japan

**Keywords:** *Aegilops umbellulata*, *Aegilops tauschii*, Barley, DNA markers, RNA sequencing, Synteny

## Abstract

**Background:**

*Aegilops umbellulata* Zhuk. (2n = 14), a wild diploid wheat relative, has been the source of trait improvement in wheat breeding. Intraspecific genetic variation of *Ae. umbellulata*, however, has not been well studied and the genomic information in this species is limited.

**Results:**

To develop novel genetic markers distributed over all chromosomes of *Ae. umbellulata* and to evaluate its genetic diversity, we performed RNA sequencing of 12 representative accessions and reconstructed transcripts by de novo assembly of reads for each accession. A large number of single nucleotide polymorphisms (SNPs) and insertions/deletions (indels) were obtained and anchored to the pseudomolecules of *Ae. tauschii* and barley (*Hordeum vulgare* L.), which were regarded as virtual chromosomes of *Ae. umbellulata*. Interestingly, genetic diversity in *Ae. umbellulata* was higher than in *Ae. tauschii,* despite the narrow habitat of *Ae. umbellulata*. Comparative analyses of nucleotide polymorphisms between *Ae. umbellulata* and *Ae. tauschii* revealed no clear lineage differentiation and existence of alleles with rarer frequencies predominantly in *Ae. umbellulata*, with patterns clearly distinct from those in *Ae. tauschii*.

**Conclusions:**

The anchored SNPs, covering all chromosomes, provide sufficient genetic markers between *Ae. umbellulata* accessions. The alleles with rarer frequencies might be the main source of the high genetic diversity in *Ae. umbellulata*.

**Electronic supplementary material:**

The online version of this article (10.1186/s12870-018-1498-8) contains supplementary material, which is available to authorized users.

## Background

*Aegilops umbellulata* Zhuk. (2n = 14), a wild diploid wheat relative, is distributed in West Asia and is known as the U-genome donor of *Ae. columnaris* and *Ae. triaristata* [1, 2]. *Ae. umbellulata* (UU genome) has crossability with tetraploid wheat (*T. turgidum* L.; AABB genome), which allows generation of synthetic hexaploids (AABBUU genome) through ABU triploids. Some combinations of interspecific crosses between *Ae. umbellulata* accessions and tetraploid wheat result in hybrid incompatibility, such as severe growth abortion and grass-clump dwarfness [3]. This observation suggests the existence of unrevealed genetic polymorphisms in *Ae. umbellulata* that potentially vary phenotypic traits.

*Ae. umbellulata* have been used for breeding of bread wheat and is a considerable resource of disease resistance genes [4–10]. Leaf rust and stripe rust resistance genes [6, 8, 11] and high-molecular weight glutenin subunits [5, 12] have been introduced into bread wheat cultivars. Chhuneja et al. (2008) [6] and Bansal et al. (2017) [8] established introgression lines of leaf and stripe rust resistance genes derived from synthetic hexaploids (AABBUU). The cross of the synthetic hexaploids (AABBUU) with *T. aestivum* cv. Chinese Spring *Ph*^*I*^, which carries an epistatic inhibitor of *Ph1* gene, induced homologous pairing and resulted in transfer of the leaf and stripe rust resistance genes of *Ae. umbellulata* into the bread wheat *T. aestivum*. Although *Ae. umbellulata* provides valuable genetic resources for breeding of bread wheat, it has not been well studied and information on its genome is limited. Evaluation of intraspecific genetic diversity based on genome-wide polymorphisms in *Ae. umbellulata* would impart practical information for designing genetic markers, facilitating the efficient use of *Ae. umbellulata* for breeding.

Since the tribe Triticeae has a large genome, most of which is occupied by repetitive sequences, development of high-quality physical maps and whole genome sequencing are challenging. RNA sequencing (RNA-seq) is one of the solutions for detection of single nucleotide polymorphisms (SNPs) and evaluation of genetic diversity by avoiding these genome complexities of the Triticeae. RNA-seq approaches for identifying novel genetic markers have been applied to several Triticeae species such as *T. monococcum* [13] and *Ae. tauschii* [14–16]. RNA-seq has the advantage of direct detection of SNPs linked to causal genes for targeted phenotypes. RNA-seq-based bulked segregant analysis narrowed down the genome location of a wheat yellow rust resistance gene, *Yr15*, and a wheat spot blotch resistance gene, *Sb3*, within 0.77 cM and 0.15 cM intervals, respectively [17, 18].

Recently, the highest-quality genome sequences have been developed in the diploid Triticeae species barley (*Hordeum vulgare* L.) [19, 20] and *Ae. tauschii* [21, 22]. By utilizing highly conserved chromosomal synteny across Triticeae species [23, 24], the pseudomolecules of barley and *Ae. tauschii* can be regarded as virtual chromosomes of other Triticeae species. By combining RNA-seq with positional information from this synteny, a large number of SNPs and indels can be anchored to the chromosomes*,* facilitating design of genome-wide genetic markers [16]. The RNA-seq-based approach for marker development is considered applicable to other wild wheat species when enough genomic information is lacking.

Here, to evaluate genetic polymorphisms and capture genetic markers in *Ae. umbellulata,* transcripts of 12 representative accessions of *Ae. umbellulata* were first reconstructed by de novo assembly of reads from RNA-seq on the Illumina MiSeq platform. Using the deduced transcript sequences, a large number of SNPs and indels between the *Ae. umbellulata* accessions were detected and anchored to the barley and *Ae. tauschii* pseudomolecules. Comparative analysis of DNA polymorphisms between *Ae. umbellulata* and *Ae. tauschii* revealed relatively high genetic diversity in *Ae. umbellulata*.

## Methods

### Plant materials, library construction and RNA sequencing

**Fig. 1:**
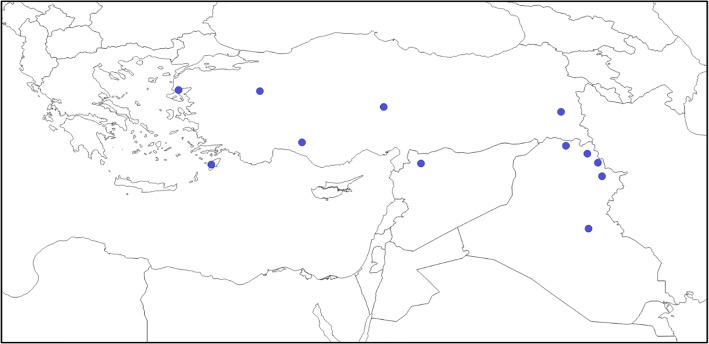
The geographic distribution of the 12 tested accessions of *Ae. umbellulata* on the map of the northwestern part of the Middle East

Twelve accessions of *Ae. umbellulata* were chosen from the wheat genetic resources database of the National BioResource Project-Wheat (Japan, https://shigen.nig.ac.jp/wheat/komugi/top/top.jsp) to represent the diversity of this species (Fig. 1; Table 1). *T. urartu* KU-199-5 was used as the outgroup species for the comparative analysis between *Ae. umbellulata* and *Ae. tauschii*. Total RNA was extracted from leaves of *Ae. umbellulata* and *T. urartu* at the seedling stage using a Sepasol-RNA I Super G solution (Nacalai Tesque, Kyoto, Japan). The total RNA was treated with DNase I at 37 °C for 20 min to remove contaminating DNA. A total of 6 to 10 μg of RNA was used for constructing paired-end libraries. The libraries were constructed with TruSeq RNA Library Preparation Kit v2 (Illumina, San Diego, CA, USA) according to the manufacturer’s instructions, and were sequenced with 300-bp paired-end reads on an Illumina MiSeq sequencer.

**Table 1:** List of *Ae. umbellulata* accessions used in this study

Accession number	Origins	Locality
KU-4017	Iraq	18.8 km NNE from Sulaymaniyah to Chuarta
KU-4026	Iraq	25.9 km S from Kirkuk to Baghdad
KU-4035	Iraq	5.5 km ENE from Koi Sanjak to Ranya
KU-4043	Iraq	SSW of Rowanduz
KU-4052	Iraq	4.4 km NW from Amadiyah Mazorka Gorge
KU-4103	Turkey	North of Van
KU-5934	Turkey	Suburbs of Kayseri
KU-5954	Turkey	Suburbs of Kutahia
KU-8-7	Turkey	Suburbs of Burdur (D)
KU-12180	Greece	5.1 km W from Platania to Laerma Rhodes
KU-12198	Greece	5.4 km E from Mithymna to Madamados Lesbos
KU-8-5	Syria	6 km W of Qatana (Damascus - Mt. Hermon)

### De novo assembly of reads from RNA-seq

Low-quality bases (average quality score per 4 bp < 30), adapter sequences, and reads < 100 bp were removed using the Trimmomatic version 0.33 tool [25]. The paired-end reads were assembled with Trinity version 2.0.6 software to reconstruct transcripts for each accession [26, 27]. If a gene had multiple isoforms, the first transcript sequence designated by Trinity was chosen as a unigene. A set of unigenes was made for each accession according to our previous report [16], and was used as a reference transcript dataset. Paired-end reads from each accession were aligned to the reference transcripts using the Bowtie 2 [28]. SAMtools and Coval software were used for SNP and indel calling [29, 30]. SNPs and indels were called when over 95% of the aligned sequences were different from those of the reference transcripts at positions with read depth > 10. Sequence data have been deposited to DDBJ Sequence Read Archive DRA006404.

### Mapping the assembled transcripts, SNPs and indels to barley and *Ae. tauschii* genome sequences

The transcripts were mapped to the barley (*Hordeum vulgare* L.) reference genome “ASM32608v1 masked” [19] from the Ensembl Plants database [31] and to the *Ae. tauschii* genome “PRJNA341983” from the NCBI database [21] using Gmap software version 2014-12-31 [32] and bedtools [33]. Based on the transcripts mapping to the pseudomolecules of *Ae. tauschii* and barley, SNPs and indels were anchored to the chromosomes. The distribution of SNPs and indels on barley and *Ae. tauschii* chromosomes were visualized using CIRCOS software [34] (Krzywinski et al. 2009).

### Development of markers and genotyping

Indel markers were designed using indels longer than 3 bp that were anchored to the barley chromosomes. Primer sets were constructed with Primer3plus software [35]. To validate marker alleles, we genotyped F_1_ hybrid from a cross between *Ae. umbellulata* accessions KU-4017 and KU-4043. Total DNA was extracted from leaves of F_1_ plants and their parents. PCR was conducted using Quick Taq HD DyeMix (TOYOBO, Osaka, Japan). PCR products were resolved in 17% acrylamide gels, and the products were visualized under UV light after staining by ethidium bromide.

### Comparison of genetic diversity between *Ae. umbellulata* and *Ae. tauschii*

The RNA-seq reads from the 10 *Ae. tauschii* accessions from the Transcriptome Shotgun Assembly division of DDBJ BioProject PRJDB4683 [16] were used for comparative analyses. We used the transcript sequences of *Ae. tauschii* KU-2075, which were constructed in our previous report [16], and *Ae. umbellulata* KU-4017 as reference transcripts. Quality control for the reads of *Ae. tauschii* and *T. urartu* was performed using Trimmomatic version 0.33 [25] in the same way as for *Ae. umbellulata*. The reads were aligned to the reference transcripts of *Ae. tauschii* KU-2075 and *Ae. umbellulata* KU-4017 using Bowtie 2 [28]. SNP calling was performed with SAMtools and Coval [29, 30] using the same criteria described above. SNPs that were assured of read depth > 10 and no ambiguous nucleotides in any accessions were selected as high-confidence SNPs and used for analyzing intra- and interspecific variation. The number of segregating sites, Tajima’s D statistic [36], and fixed nucleotide differences between species were estimated with DnaSP v5 software [37]. A neighbor-joining tree and a maximum likelihood tree were constructed based on the high-confidence SNPs. Bootstrap probability was calculated for 1000 replications.

### Estimation of orthologous transcripts of *Ae. umbellulata* and *Ae. tauschii*

Orthologous pairs of the reference transcripts of *Ae. tauschii* KU-2075 and *Ae. umbellulata* KU-4017 were estimated according to reciprocal best hits of BLAST analysis. A BLASTN search was performed using transcripts of *Ae. tauschii* KU-2075 as the queries against transcripts of *Ae. umbellulata* KU-4017, and vice versa. When the same best hit was detected and query coverage was over 80% in both BLAST analyses, the transcripts from *Ae. umbellulata* KU-4017 and *Ae. tauschii* KU-2075 were judged an orthologous pair.

### Gene expression analysis

The mapped reads that were concordantly aligned to the reference transcripts were chosen from the alignment file with SAMtools [29]. Fragments per kilobase per million mapped reads (FPKM) values were calculated based on the concordantly mapped reads [38].

## Results

### RNA sequencing of 12 *Ae. umbellulata* accessions

To evaluate genetic diversity based on a large number of DNA polymorphisms in the U-genome species *Ae. umbellulata*, RNA-seq was performed on the 12 representative accessions, generating 3.5–6.1 million paired-end reads per one accession (Table 2). These reads were analyzed according to the workflow shown in Additional file 1: Figure S1. After filtering out reads with low quality, 2.2–3.9 million paired-end reads (56.2–74.1%) were obtained. Due to the absence of a reference genome for *Ae. umbellulata*, transcript sequences for each of the 12 accessions were constructed by de novo assembly of the filtered reads. For each accession, 20,996 to 59,253 transcripts with N50 values of 899 to 1365 bp were deduced. One isoform was chosen as a unigene if a transcript had multiple isoforms. Finally, 12 sets of unigenes composed of 20,675 to 55,831 representative isoforms were obtained (Table 2) and used as reference transcript datasets for pairwise alignments between the accessions.

**Table 2:** Summary of RNA sequencing for 12 accessions of *Ae. umbellulata*

Accession	Read pairs	Filtered read pairs (%^a^)	(unigenes)	N50 (bp)	Median contig length (bp)	Total assembled bases (Mbp)
KU-4017	3,738,403	2,515,683 (67.29%)	39,359 (37640)	1238	658	36
KU-4026	4,935,634	3,429,252 (69.48%)	20,996 (20675)	900	578.5	15.6
KU-4035	6,077,268	3,890,911 (64.02%)	57,029 (52216)	1350	654	52.9
KU-4043	4,090,151	2,392,708 (58.50%)	50,985 (48590)	1095	518	38.9
KU-4052	3,829,992	2,152,503 (56.20%)	47,320 (44869)	1179	577	39.4
KU-4103	3,875,477	2,506,545 (64.68%)	31,418 (30873)	899	507	22.1
KU-5934	5,114,283	3,507,234 (68.58%)	57,466 (52751)	1332	630	52.7
KU-5954	3,686,807	2,694,916 (73.10%)	45,164 (41780)	1365	699	44.1
KU-12180	3,455,952	2,560,779 (74.10%)	46,323 (44000)	1229	631	41.2
KU-12198	3,623,492	2,666,981 (73.60%)	50,069 (46178)	1359	655	47,7
KU-8-5	3,669,766	2,699,452 (73.56%)	52,648 (48981)	1259	654	48.2
KU-8-7	3,798,153	2,524,378 (66.46%)	59,253 (55831)	1160	524	46.9

^a^Percentage of the number of filtered read pairs per the number of read pairs

### Genome-wide identification of SNPs and indels in *Ae. umbellulata*

To detect SNPs and indels among the accessions, the filtered reads of each accession were aligned to the reference transcripts of all other accessions, and SNPs and indels were called according to the thresholds with read depth > 10. SNPs and indels identified from comparisons of the same accessions were regarded as artifacts. After filtering to remove these putative artifacts, 2925–44,751 SNPs and 77–1389 indels were obtained among the accessions (Table 3). The maximum numbers of SNPs and indels were obtained between KU-4035 and KU-12180 (44,751 SNPs and 1389 indels), with the minimum between KU-4017 and KU-4026 (2925 SNPs and 77 indels).

**Table 3:** The number of SNPs and indels detected in each transcript-read pairing of 12 *Ae. umbellulata* accessions

Transcript model	Read												total NR SNPs and indels
	KU- 4017	KU- 4026	KU- 4035	KU- 4043	KU- 4052	KU- 4103	KU- 5934	KU- 5954	KU- 12,180	KU- 12,198	KU- 8–5	KU- 8–7	
KU-		2925	16,611	11,171	12,091	5316	24,975	14,064	29,726	23,244	13,400	24,601	85,758
4017		77	524	348	381	169	854	401	869	648	443	846	3284
KU-	10,754		16,535	11,666	12,869	5428	19,463	11,889	23,915	17,452	11,478	20,185	63,233
4026	350		612	429	420	153	762	405	787	565	412	754	2645
KU-	9069	3639		11,969	13,141	5372	25,450	14,991	30,925	23,575	13,356	25,797	89,369
4035	274	94		426	423	167	889	387	901	651	396	847	3303
KU-	9748	2959	17,571		11,772	5325	24,871	15,017	30,191	22,942	13,557	26,117	85,656
4043	287	83	611		404	155	867	423	883	662	459	809	3398
KU-	10,355	3393	20,370	11,585		5172	25,332	14,713	30,020	23,340	14,824	26,197	88,716
4052	347	92	665	408		153	848	406	951	716	493	872	3525
KU-	11,611	3811	19,020	13,758	13,022		21,807	13,405	25,741	19,851	14,038	21,764	71,071
4103	348	99	672	462	429		827	417	786	605	474	732	2932
KU-	14,265	3465	25,574	16,432	16,766	6131		13,619	31,109	23,359	15,968	25,591	89,344
5934	443	97	854	582	527	187		385	932	663	540	855	3489
KU-	15,032	3585	28,423	18,003	18,391	6424	25,505		30,219	22,205	17,868	25,394	91,059
5954	433	89	909	572	560	174	859		942	650	563	872	3723
KU-	24,232	6057	44,751	27,569	29,176	9695	42,984	27,022		24,142	30,721	34,445	99,411
12,180	698	163	1389	901	896	296	1384	768		719	935	1140	4246
KU-	21,096	5282	40,683	24,658	25,784	9259	39,208	22,641	26,570		27,036	31,815	100,683
12,198	614	117	1311	777	805	246	1263	627	809		813	1022	4085
KU-	12,822	3444	23,775	15,520	16,626	5893	24,993	15,271	30,526	23,495		25,746	91,080
8–5	375	100	784	558	577	164	907	439	952	689		860	3635
KU-	17,577	5041	33,501	20,936	21,374	7235	32,035	18,925	30,559	24,589	21,646		94,187
8–7	530	128	1068	694	650	227	1057	557	924	716	670		3728

Upper and lower numbers at each comparison indicate SNPs and indels, respectively. *NR* non-redundant

**Fig. 2:**
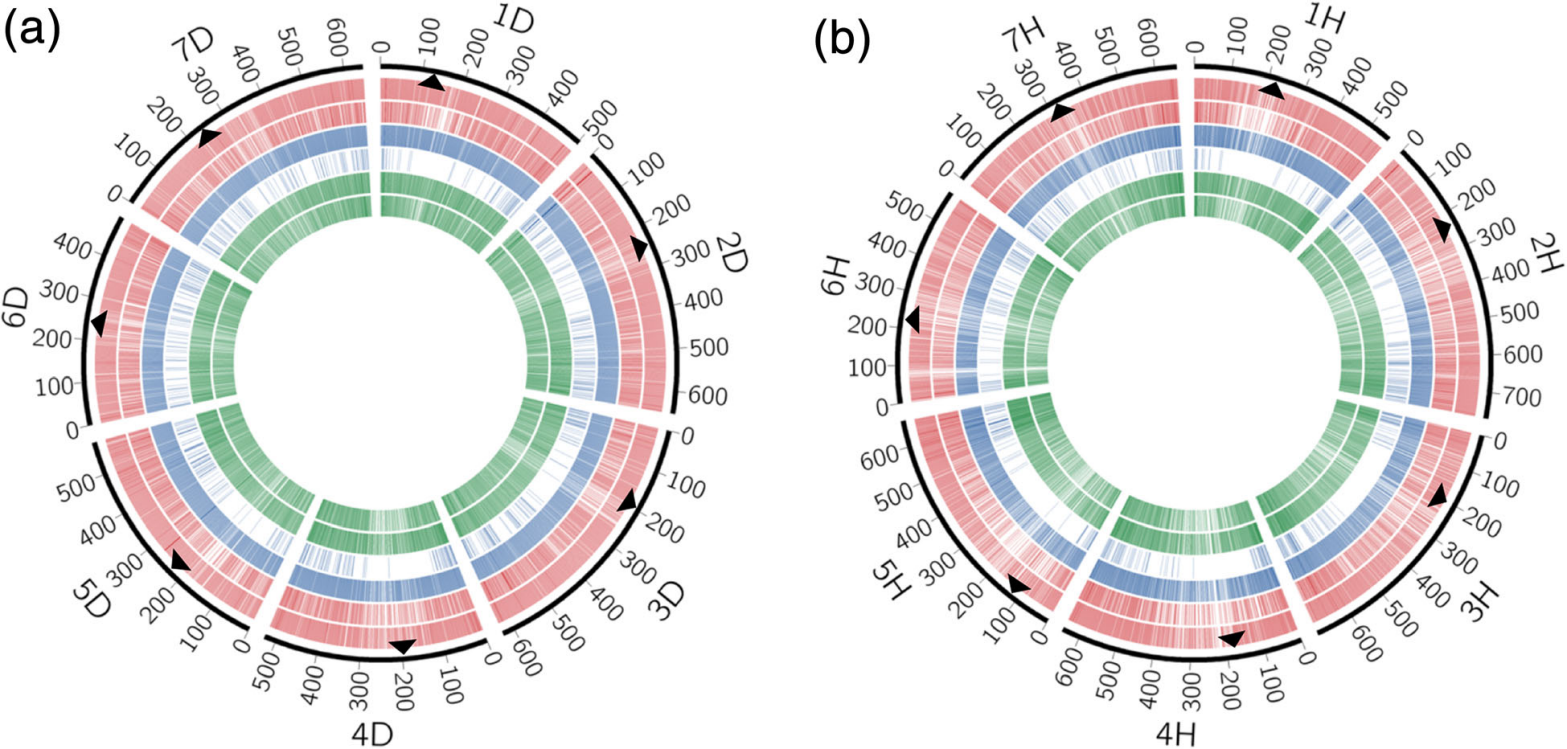
Distribution of transcripts and SNPs detected in pairwise comparisons of *Ae. umbellulata* accessions on the physical map of *Ae. tauschii* (**a**) and barley (**b**); scale in Mb. The three circles of the same color show the number of transcripts and SNPs from outer to inner circles. The red circles indicate the richest SNP pairs (KU-4035 read-mapped to KU-12180 transcript). The blue circles indicate the least rich pairs (KU-4026 read-mapped to KU-4017 transcript) in each combination of lineages. The green circles indicate the non-redundant SNPs mapped against KU-4043 transcripts. Arrowheads represent centromeric positions of each chromosome

For efficient use of the identified SNPs and indels as genetic markers, their chromosomal locations must be known. Here, we used the *Ae. tauschii* and barley pseudomolecules as virtual chromosomes of *Ae. umbellulata*, and mapped the unigene sequences of the *Ae. umbellulata* reference transcript datasets to the *Ae. tauschii* and barley chromosomes. In the reference transcripts, 75.87–85.35% of the unigenes were mapped to *Ae. tauschii* and 52.08–67.69% to barley chromosomes (Additional file 1: Table S1). Based on the positional information of the mapped unigenes, SNPs and indels were anchored to the chromosomes of both species. In any pairwise comparison between *Ae. umbellulata* accessions, 81.83–89.50% of SNPs and 75.28–89.26% of indels were anchored to *Ae. tauschii* chromosomes, while 63.17–75.16% of SNPs and 59.04–77.78% of indels were anchored to barley chromosomes (Additional file 1: Tables S2, S3). The distribution of SNPs over each chromosome of *Ae. tauschii* and barley was visualized with CIRCOS [34] for the *Ae. umbellulata* accession pairs with the maximum or minimum number of SNPs. The SNPs covered all chromosomes (Fig. 2).

**Fig. 3:**
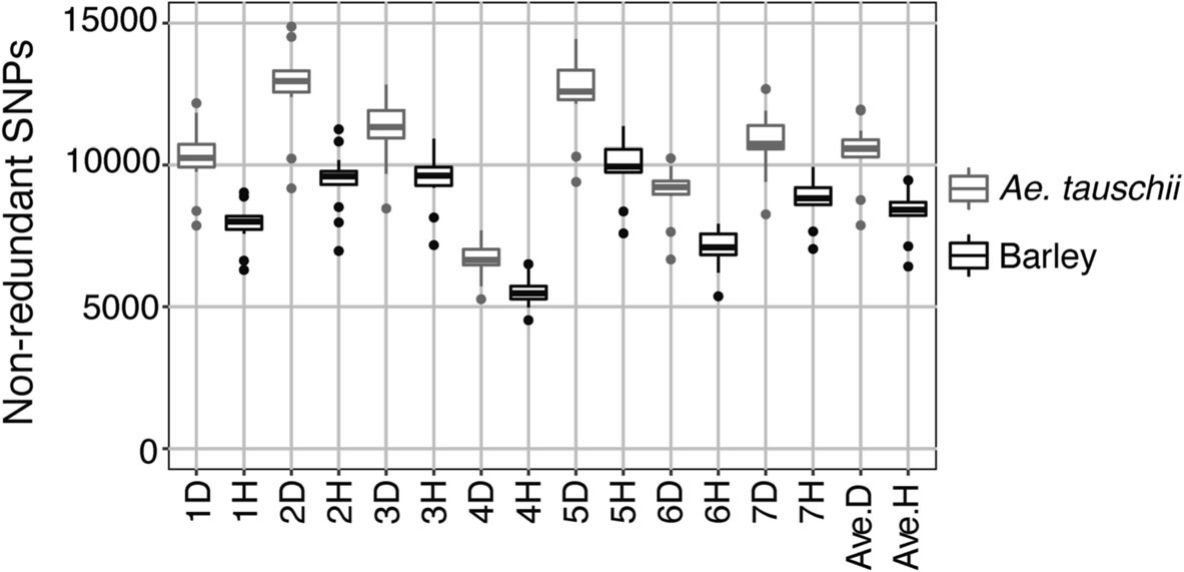
Boxplots of non-redundant SNPs anchored to chromosomes of *Ae. tauschii* (gray) and barley (black). The number of non-redundant SNPs on the chromosomes for each reference transcript dataset is plotted. Ave.D and Ave.H respectively indicate average number of non-redundant SNPs per chromosome when genomes of *Ae. tauschii* and barley were used

Non-redundant SNPs and indels were estimated for each of the 12 sets of reference transcripts. A total of 63,233–100,683 non-redundant SNPs and 2645–4246 non-redundant indels were detected in the tested *Ae. umbellulata* accessions (Table 3). On average, 73,075 non-redundant SNPs (85.07%) were anchored to *Ae. tauschii* chromosomes, and 58,247 (70.40%) non-redundant SNPs to barley chromosomes (Additional file 1: Tables S4, S5). The smallest number of anchored non-redundant SNPs was observed on chromosomes 4D in *Ae. tauschii* and 4H in barley (Fig. 3). Each chromosome of *Ae. tauschii* and barley had an average of 10,439 and 8321 non-redundant SNPs, respectively. The anchored non-redundant SNPs were distributed over all seven chromosomes of *Ae. tauschii* and barley (Fig. 2).

**Fig. 4:**
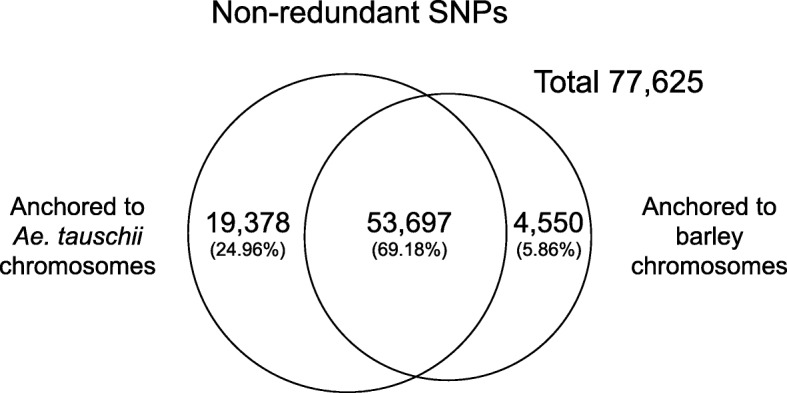
Venn diagrams of non-redundant SNPs anchored to the *Ae. tauschii* and barley chromosomes. The numbers indicate mean values of non-redundant SNPs and indels derived from each of the reference transcript datasets

We estimated the percentages of non-redundant SNPs anchored to the *Ae. tauschii* chromosomes overlapped those on barley chromosomes (Fig. 4). Venn diagrams showed that 69.18% of non-redundant SNPs were anchored to both *Ae. tauschii* and barley chromosomes. The percentage of non-redundant SNPs uniquely anchoring to *Ae. tauschii* chromosomes was 24.96%. Only 5.86% of non-redundant SNPs were uniquely anchored to barley chromosomes. After integration of these anchored non-redundant SNPs, 77,625 non-redundant SNPs were placed on the chromosomes.

### Application of indel markers to confirmation of F_1_ formation

To confirm usefulness of the identified polymorphisms as genetic markers, primer sets for 27 indels were designed. The indel markers were applied to genotype F_1_ hybrid from a cross between two *Ae. umbellulata* accessions, KU-4017 and KU-4043; nine markers enabled detection of the genetic differences between the accessions and confirmed their F_1_ formation (Additional file 1: Figure S2). The difference in amplicon size between the parents was observed in the five markers. Presence/absence of amplicons between the parents was detected in the two markers. In the other two markers, the parents were distinguished by an extra band.

### Comparison of genetic diversity in *Ae. umbellulata* and *Ae. tauschii*

**Fig. 5:**
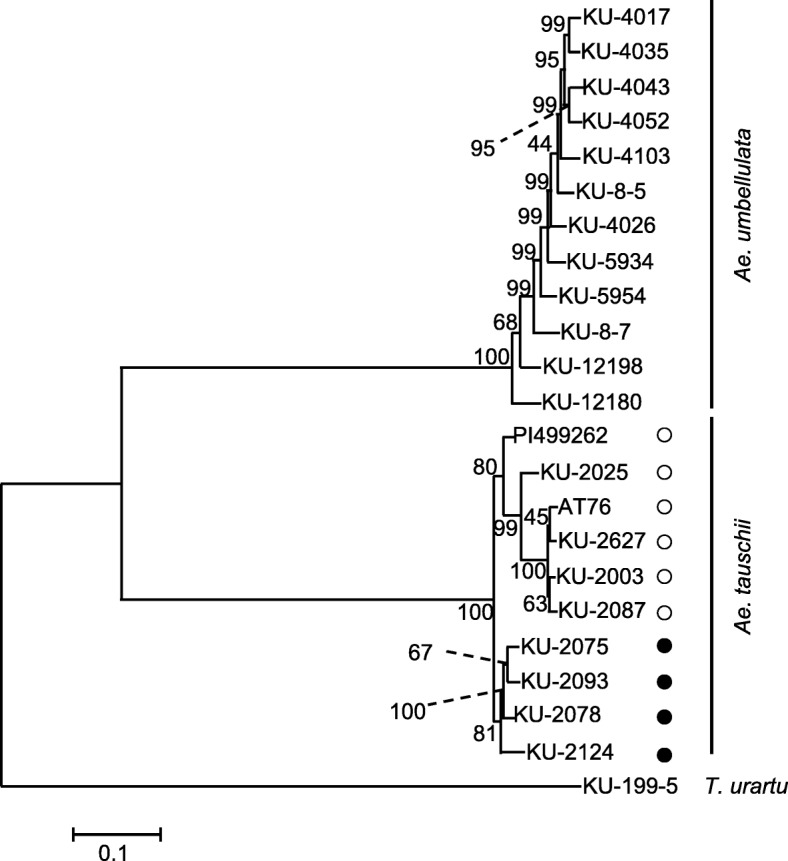
Phylogenetic relationship between the 12 *Ae. umbellulata* accessions, 10 *Ae. tauschii* accessions and one *T. urartu* accession based on SNPs estimated using the *Ae. umbellulata* KU-4017 reference transcript dataset. The tree was constructed by the maximum-likelihood method. The bootstrap values (1000 replicates) are shown on each branch. White and black circles of *Ae. tauschii* respectively correspond to the divergent lineages TauL1 and TauL2 [39]. *T. urartu* was used as the outgroup species

*Ae. tauschii* is widely distributed over central Eurasia and has three divergent lineages, TauL1, TauL2 and TauL3 [39]. On the other hand, the habitat of *Ae. umbellulata* is limited to West Asia. To examine how differences in geographic distribution and evolutionary history of these species affected the extent of DNA polymorphisms and the distribution of allele frequency, genetic diversity in *Ae. umbellulata* and *Ae. tauschii* was evaluated with SNPs deduced using the same RNA-seq platform. To compare intraspecific diversity of the two *Aegilops* species, reads from RNA-seq of the 12 *Ae. umbellulata* accessions, the 10 *Ae. tauschii* accessions [16] and *T. urartu* KU-199-5 were aligned to the reference transcripts of *Ae. umbellulata* KU-4017. *T. urartu* KU-199-5 was used as an outgroup species. To elucidate the phylogenetic relationship of *Ae. umbellulata* and *Ae. tauschii* accessions, maximum likelihood and neighbor-joining trees were constructed based on the high-confidence SNPs (Fig. 5; Additional file 1: Figure S3a). The three species were clearly separated, with the *Aegilops* species more closely related than *T. urartu*, with fixed nucleotide differences between *Ae. umbellulata* and *Ae. tauschii* smaller than those between *Ae. tauschii* and *T. urartu* or between *Ae. umbellulata* and *T. urartu* (Additional file 1: Table S6). The external branches of *Ae. umbellulata* were longer than those of *Ae. tauschii*. *Ae. umbellulata* KU-12180 was isolated from the other accessions, supporting observations from the phylogenetic trees constructed based on nucleotide polymorphisms in a small number of genes [3]. However, the clear divergent lineages observed in *Ae. tauschii* were not found in the *Ae. umbellulata* accessions (Fig. 5)*.* When the reference transcripts of *Ae. tauschii* KU-2075 was used for the alignments and SNP calling, similar results were obtained (Additional file 1: Table S6, Figures S3b, S4).

**Fig. 6:**
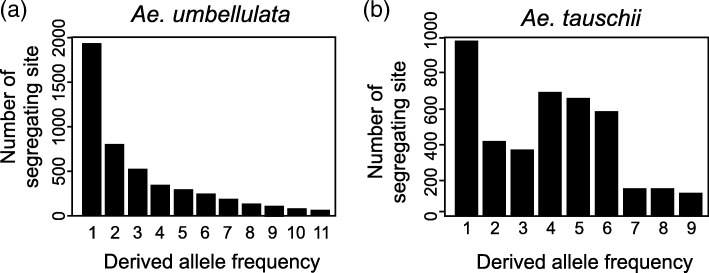
Derived allele frequency distribution in *Ae. umbellulata* (*n* = 12) (**a**) and *Ae. tauschii* (*n* = 10) (**b**), respectively. *Ae. umbellulata* KU-4017 transcripts were used as the reference. Derived alleles were estimated using the outgroup species *T. urartu*

The number of segregating sites in *Ae. umbellulata* was larger than in *Ae. tauschii* (Table 4), indicating that *Ae. umbellulata* has relatively high genetic diversity. To test how differences in habitat and evolutionary history between *Ae. umbellulata* and *Ae. tauschii* affected allele frequency distribution in these two species, the derived allele frequency distribution for each species was estimated using *T. urartu* as an outgroup species (Fig. 6). At a polymorphic site, a nucleotide that is inconsistent with that of outgroup species is defined as a derived allele, because this allele is considered to be newly generated by a mutation in population of the tested species [40]. The derived allele frequency distributions of the two species showed distinct patterns. Alleles with intermediate frequency were predominantly detected in *Ae. tauschii*, while alleles with rarer frequency were more common in *Ae. umbellulata*. As expected from the difference in the allele frequency distributions, Tajima’s D statistic [36] for *Ae. tauschii* and *Ae. umbellulata* respectively gave positive and negative values (Table 4).

**Table 4:** Summary of nucleotide polymorphisms in *Ae. umbellulata* and *Ae. tauschii*

Reference	*Ae. umbellulata* KU-4017		*Ae. tauschii* KU-2075	
SNPs	*Ae. umbellulata*	*Ae. tauschii*	*Ae. umbellulata*	*Ae. tauschii*
# of accession	12	10	12	10
# of site	31,677	31,677	29,702	29,702
# of segregating site	4751	4136	4301	3832
singleton	1992	1103	1759	1019
non-singleton	2759	3033	2542	2813
Tajima’s D	−0.24 NS	0.86 NS	−0.22 NS	0.89 NS

**Fig. 7:**
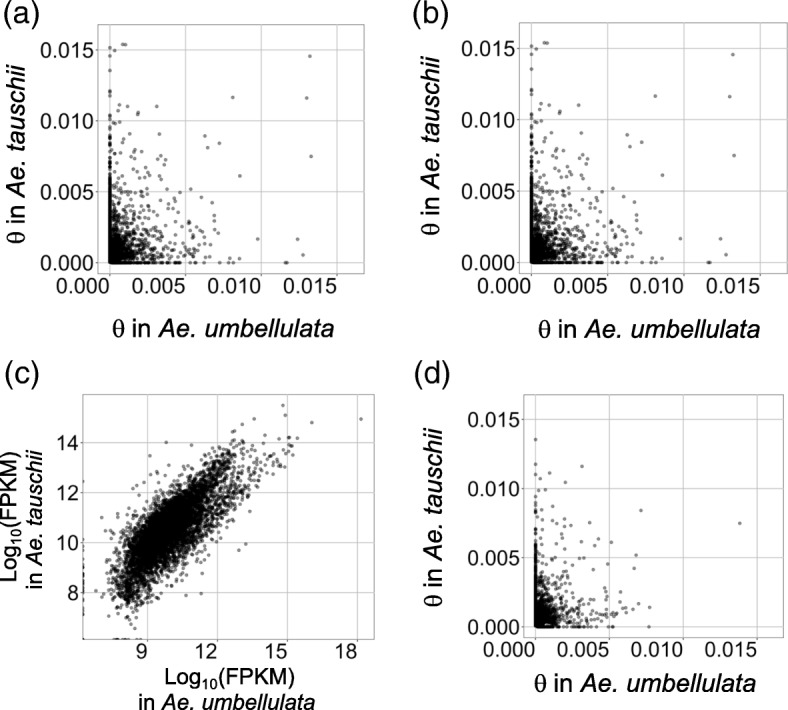
Scatter plot of nucleotide diversity (θ)in *Ae. umbellulata* (n = 12) and *Ae. tauschii* (n = 10) when 7666 transcripts derived from *Ae. umbellulata* KU-4017 were used as the references (**a**) and when 6622 transcripts derived from *Ae. tauschii* KU-2075 were used as the references (**b**). For both sets of transcripts, at least one of the species had polymorphisms. Scatter plot of gene expression (FPKM) in the 6062 orthologous pairs of *Ae. umbellulata* KU-4017 and *Ae. tauschii* KU-2075 retrieved by reciprocal best hits of BLAST analyses (**c**). Scatter plot of θ in the 3354 orthologous pairs of *Ae. umbellulata* KU-4017 and *Ae. tauschii* KU-2075 that had polymorphisms in one of the species (**d**). Kendall’s rank correlation (τ) for (**a**), (**b**), (**c**), and (**d**) was − 0.026 (*p* = 0.0033), 0.043 (*p* = 5.3e-6), 0.577 (*p* < 2.2e-16) and 0.049 (*p* = 0.0002), respectively

Nucleotide diversity (θ) [41] in *Ae. umbellulata* and *Ae. tauschii* was estimated for each transcript. The θ value for each transcript of *Ae. umbellulata* was weakly correlated with that of *Ae. tauschii* (Figs. 7a, b: Kendall’s rank correlation τ = − 0.026 and 0.043). To avoid the possibility of bias due to differences in the accuracy and efficiency of short read alignments between intra- and interspecies, we compared θ for the 6062 orthologous pairs between *Ae. umbellulata* and *Ae. tauschii*. These pairs were retrieved by reciprocal best hits of BLAST analysis between the reference transcript datasets of *Ae. umbellulata* KU-4017 and *Ae. tauschii* KU-2075. This approach enables evaluation of genetic diversity using only the θ value based on SNPs derived from the intraspecies alignments of reads. Although gene expression of the orthologous pairs showed a relatively strong correlation (Fig. 7c: τ = 0.577), the values of θ between the pairs designated a weak correlation (Fig. 7d: τ = 0.049). Taken together, the reproducible observations from different approaches underpin the distinct extent of nucleotide polymorphisms between *Ae. umbellulata* and *Ae. tauschii* at the gene level.

## Discussion

### RNA-seq is a powerful approach to identify novel genetic markers for Triticeae species

To identify genome wide polymorphisms (SNPs and indels) and to develop novel genetic markers, we conducted 300-bp paired-end RNA sequencing of leaf tissues from 12 representative *Ae. umbellulata* accessions using the Illumina MiSeq platform. By using *Ae. tauschii* and barley pseudomolecules as the virtual chromosomes of *Ae. umbellulata* due to the conserved synteny between Triticeae species [23, 24], an average of 73,075 and 58,247 non-redundant SNPs in *Ae. umbellulata* were successfully anchored to the chromosomes of *Ae. tauschii* and barley, respectively (Fig. 3; Additional file 1: Tables S4, S5). The application of reference-quality genome sequences of *Ae. tauschii* [21] dramatically improved the number of SNPs anchored to the chromosomes compared with a previous study [16], in which SNPs in *Ae. tauschii* were linked to the chromosomes by combining the draft genome sequences of *Ae. tauschii* [42] with its genetic linkage map [43]. Even when SNPs in *Ae. tauschii* were mapped to *Ae. tauschii* chromosomes, the number of anchored SNPs was slightly smaller than when the SNPs were mapped to the chromosomes of barley with reference-quality genome sequences [16]. The elaboration of SNP anchoring enabled capturing an average of 10,439 non-redundant SNPs per chromosome (Fig. 3; Additional file 1: Table S5), which were well distributed over each chromosome (Fig. 2). Since polymorphisms derived RNA-seq data were composed of only SNPs and indels in exons and untranslated regions of the expressed genes, the RNA-seq-based approach avoided the repetitiveness of intergenic regions and much of the genome complexity, resulting in identification of a large number of SNPs anchored to the virtual chromosomes. Recently, a high-density consensus linkage map including 3009 SNP markers derived from genotyping-by-sequencing was constructed in two biparental populations from four accessions of *Ae. umbellulata* [9]. The RNA-seq approach fills the gaps left by other genotyping methods such as genotyping-by-sequencing when developing genetic markers for Triticeae species without a genome sequence, such as *Ae. umbellulata*.

In our RNA-seq-based approach, the identified SNPs and indels were arranged on the *Ae. umbellulata* chromosomes in an order reflecting the conserved synteny with *Ae. tauschii* and barley (Fig. 2). When a genetic map is constructed using these anchored SNPs and indels, changes in the marker order should be considered carefully due to the existence of chromosomal rearrangements in *Ae. umbellulata*. Structural rearrangements have been observed for *Ae. umbellulata* chromosomes when the order of genetic markers was compared among *Ae. umbellulata*, *Ae. tauschii* and common wheat [9, 44–46]. For example, chromosome 4 U has segmental homoeology to the group 6 chromosomes of common wheat [46]. Similarly, partial segments of chromosome 6 U have homoeology to hexaploid wheat group 4 and 5 chromosomes [9]. These observations support the occurrence of structural rearrangements such as translocation in *Ae. umbellulata*.

The power of indel detection with RNA-seq is not as high as that of SNPs, because indels in exons often have functionally deleterious effects on proteins and are purged from the genome by purifying selection. Notwithstanding this disadvantage, RNA-seq still provides useful indel markers for genetic mapping [47]. The indel markers were effective for validating detection of F_1_ alleles between *Ae. umbellulata* KU-4017 and KU-4043 (Additional file 1: Figure S2). These markers would allow rough map construction.

### Contrasting patterns of nucleotide diversity between *Ae. umbellulata* and *Ae. tauschii*

Differences in the habitats, morphology, population structure and phenological traits between *Ae. tauschii* and *Ae. umbellulata* may result in differences in the pressures of natural selection and the effect of genetic drift on genes, shaping the extent of DNA polymorphisms and allele frequency distribution between the species. In spite of the limited habitats of *Ae. umbellulata*, the present study showed that *Ae. umbellulata* has higher genetic diversity than the more widely distributed species *Ae. tauschii* (Fig. 5; Table 4). This observation is consistent with a previous report [48], in which intra- and interspecific genetic variation in seven diploid *Aegilops* species was evaluated using amplified fragment length polymorphisms, also concluding that genetic diversity in *Ae. umbellulata* is higher than in *Ae. tauschii*. Our comparative analyses showed no clear lineage differentiation in *Ae. umbellulata* (Fig. 5; Additional file 1: Figures S3, S4) and the prevalence of alleles with rarer frequencies (Fig. 6; Additional file 1: Figure S5), implying that the alleles with rarer frequencies are the main source of the genetic diversity observed in *Ae. umbellulata*.

The longer external branches of the phylogenetic tree in *Ae. umbellulata* suggest higher genetic differentiation of each *Ae. umbellulata* accession than *Ae. tauschii* (Fig. 5; Additional file 1: Figures S3, S4). Generally self-pollination inhibits gene flow via pollen, increasing genetic differentiation among local populations [49]. Since *Ae. umbellulata* is a self-fertilizing plant, this general view could be applicable to the observed genetic differentiation between the accessions of *Ae. umbellulata*. Considering *Ae. tauschii* is also a self-fertilizing species, another factor may contribute to shaping the distinct patterns of nucleotide polymorphism in these two species. If the time of expansion and colonization into the modern habitats differed between species, neutral mutations are expected to have accumulated more within a local population of the species with the earlier expansion and colonization, generating genetic differentiation between local populations under the limited gene flow. If this hypothesis is accepted, the time of expansion and colonization into the modern habitat of *Ae. umbellulata* is presumed to be older than that of *Ae. tauschii*. These different evolutionary scenarios and habitats of *Ae. tauschii* and *Ae. umbellulata* are likely to have shaped distinct genetic diversity for each gene from their common ancestor. The scatter plots of nucleotide diversity in the transcripts of *Ae. umbellulata* and *Ae. tauschii* show weaker correlations between the orthologous pairs (Fig. 7), suggesting that genes of *Ae. umbellulata* were subjected to natural selection pressure and effects of genetic drift that were distinct from those of *Ae. tauschii*. Future larger-scale population genomic analyses in both species will disclose population dynamics with higher resolution and more powerfully detect footprints of natural selection in each gene.

## Conclusion

The RNA-seq-based approach is efficient for development of a large number of molecular markers and for conducting population genetic analyses for a large number of genes in wheat wild relatives such as *Ae. umbellulata* lacking genomic information. In addition, *Ae. umbellulata,* harboring relatively high genetic diversity, has considerable potential as a genetic resource for breeding of common wheat.

## Additional file

Additional file 1 : **Table S1.** Summary of the number of unigenes anchored to barley and *Ae. tauschii* genome. **Table S2.** The number of SNPs and indels anchored to the chromosomes of *Ae. tauschii* out of the SNPs and indels detected in each transcript-read pairing of 12 *Ae. umbellulata* accessions. **Table S3.** The number of SNPs and indels anchored to the barley chromosomes out of the SNPs and indels detected in each transcript-read pairing of 12 *Ae. umbellulata* accessions. **Table S4.** The number of non-redundant SNPs anchored to each *Ae. tauschii* chromosome. **Table S5.** The number of non-redundant SNPs anchored to each barley chromosome. **Table S6.** Summary of nucleotide polymorphism and divergence in *Ae. umbellulata*, *Ae. tauschii* and *T. urartu*. **Figure S1.** The workflow of RNA-seq analysis. **Figure S2.** Images of polyacrylamide gel electrophoresis for indel markers. **Figure S3.** Phylogenetic relationship between 12 *Ae. umbellulata* accessions, 10 *Ae. tauschii* accessions and one *T. urartu* accession based on SNPs that was estimated by using the *Ae. umbellulata* KU-4017 reference transcript dataset (a) and the *Ae. tauschii* KU-2075 reference transcript dataset (b). These trees were constructed by Neighbor-Joining method. **Figure S4.** Phylogenetic relationship between the 12 *Ae. umbellulata* accessions, 10 *Ae. tauschii* accessions and one *T. urartu* accession based on SNPs estimated using the *Ae. tauschii* KU-2075 reference transcript dataset. The tree was constructed by the maximum-likelihood method. **Figure S5.** Derived allele frequency distribution in *Ae. umbellulata* (*n* = 12) (a) and *Ae. tauschii* (*n* = 10) (b), respectively. *Ae. tauschii* KU-2075 transcripts were used as the reference. Derived alleles were estimated using the outgroup species *T. urartu.* (PDF 824 kb)

## Abbreviations

FPKM: Fragments per kilobase per million mapped reads
indels: Insertions and deletions
RNA-seq: RNA-sequencing
SNPs: Single nucleotide polymorphisms
