# Supplementary material for: RNA-seq analysis reveals considerable genetic diversity and provides genetic markers saturating all chromosomes in the diploid wild wheat relative Aegilops umbellulata
Source: BMC Plant Biol. 2018 Nov 8;18:271. doi: 10.1186/s12870-018-1498-8 (PMC6225718; doi:10.1186/s12870-018-1498-8)
Supplement: Supplementary file 1 — : Table S1. Summary of the number of unigenes anchored to barley and Ae. tauschii genome. Table S2. The number of SNPs and indels anchored to the chromosomes of Ae. tauschii out of the SNPs and indels detected in each transcript-read pairing of 12 Ae. umbellulata accessions. Table S3. The number of SNPs and indels anchored to the barley chromosomes out of the SNPs and indels detected in each transcript-read pairing of 12 Ae. umbellulata accessions. Table S4. The number of non-redundant SNPs anchored to each Ae. tauschii chromosome. Table S5. The number of non-redundant SNPs anchored to each barley chromosome. Table S6. Summary of nucleotide polymorphism and divergence in Ae. umbellulata, Ae. tauschii and T. urartu. Figure S1. The workflow of RNA-seq analysis. Figure S2. Images of polyacrylamide gel electrophoresis for indel markers. Figure S3. Phylogenetic relationship between 12 Ae. umbellulata accessions, 10 Ae. tauschii accessions and one T. urartu accession based on SNPs that was estimated by using the Ae. umbellulata KU-4017 reference transcript dataset (a) and the Ae. tauschii KU-2075 reference transcript dataset (b). These trees were constructed by Neighbor-Joining method. Figure S4. Phylogenetic relationship between the 12 Ae. umbellulata accessions, 10 Ae. tauschii accessions and one T. urartu accession based on SNPs estimated using the Ae. tauschii KU-2075 reference transcript dataset. The tree was constructed by the maximum-likelihood method. Figure S5. Derived allele frequency distribution in Ae. umbellulata (n = 12) (a) and Ae. tauschii (n = 10) (b), respectively. Ae. tauschii KU-2075 transcripts were used as the reference. Derived alleles were estimated using the outgroup species T. urartu. (PDF 824 kb) [file 12870_2018_1498_MOESM1_ESM.pdf]

Table S1 Summary of the number of unigenes anchored to barley and *Ae. tauschii* genome.

	Total Unigenes	Anchored to *H. vulgare* genome (%)	Anchored to *Ae. tauschii* genome (%)	Overlaps between *H.vulgare* and *Ae. tauschii* (%)	Only anchored to *H. vulgare* genome (%)	Only anchored to *Ae. tauschii* genome (%)	Total anchored unigenes (%)
KU-4017	37640	21817 (57.96)	29887 (79.40)	20385 (65.09)	1432 (4.57)	9502 (30.34)	31319 (83.21)
KU-4026	20675	13994 (67.69)	17646 (85.35)	13284 (72.37)	710 (3.87)	4362 (23.76)	18356 (88.78)
KU-4035	52116	27142 (52.08)	39577 (75.94)	24954 (59.75)	2188 (5.24)	14623 (35.01)	41765 (80.14)
KU-4043	48590	26718 (54.99)	37722 (77.63)	24883 (62.90)	1835 (4.64)	12839 (32.46)	39557 (81.41)
KU-4052	44869	24878 (55.45)	35136 (78.31)	23156 (62.82)	1722 (4.67)	11980 (32.50)	36858 (82.15)
KU-4103	30873	19191 (62.16)	25021 (81.04)	18069 (69.12)	1122 (4.29)	6952 (26.59)	26143 (84.68)
KU-5934	52751	27712 (52.53)	40024 (75.87)	25544 (60.54)	2168 (5.14)	14480 (34.32)	42192 (79.98)
KU-5954	41780	23260 (55.67)	32361 (77.46)	21562 (63.31)	1698 (4.99)	10799 (31.71)	34059 (81.52)
KU-12180	44000	24540 (55.77)	34241 (77.82)	22814 (63.43)	1726 (4.80)	11427 (31.77)	35967 (81.74)
KU-12198	46178	25223 (54.62)	35680 (77.27)	23347 (62.17)	1876 (4.06)	12333 (26.71)	37556 (81.33)
KU-8-5	48981	25779 (52.63)	37183 (75.91)	23862 (61.03)	1917 (4.90)	13321 (34.07)	39100 (79.83)
KU-8-7	55831	29410 (52.68)	42799 (76.66)	27209 (60.46)	2201 (4.89)	15590 (34.64)	45000 (80.60)
Average	43690.3	24138.7 (55.25)	33939.8 (77.68)	22422.4 (62.89)	1716.3 (4.81)	11517.3 (32.30)	35656.0 (81.61)
Median	45523.5	25050.5 (55.03)	35408 (77.78)	23251.5 (62.49)	1780.5 (4.79)	12156.5 (32.67)	37207 (81.73)

Table S2 The number of SNPs and indels anchored to the chromosomes of *Ae. tauschii* out of the SNPs and indels detected in each transcript-read pairing of 12 *Ae. umbellulata* accessions

Transcript model	Read													
	KU-4017	KU-4026	KU-4035	KU-4043	KU-4052	KU-4103	KU-5934	KU-5954	KU-12180	KU-12198	KU-8-5	KU-8-7	Total NR SNPs and indels	
KU-4017		2407	13931	9622	10313	4475	20939	11750	25835	19713	11262	21081	84349	SNP
		65	435	290	317	138	701	324	724	534	349	710	3207	indel
KU-4026	9520		14537	10244	11503	4858	17077	10452	21217	15262	10123	17864	61942	SNP
	310		541	379	373	132	675	354	692	499	356	671	2579	indel
KU-4035	7563	3034		10085	11252	4666	21248	12421	26178	19619	11002	21530	87795	SNP
	236	79		341	356	135	747	320	760	541	313	701	3227	indel
KU-4043	8416	2443	14767		10171	4717	20758	12783	26085	19586	11471	22397	83973	SNP
	245	71	485		337	136	696	332	752	559	377	665	3304	indel
KU-4052	8626	2854	16943	9866		4594	21388	12248	25944	19853	12547	22225	87230	SNP
	288	77	541	339		131	686	330	804	598	407	703	3435	indel
KU-4103	10259	3272	16431	12114	11618		18936	11601	22946	17373	12178	19218	69449	SNP
	301	85	578	401	380		711	352	697	540	412	634	2849	indel
KU-5934	12053	2948	21292	13681	14185	5183		11387	26417	19391	13199	21152	88051	SNP
	380	79	719	482	439	157		314	788	528	438	681	3412	indel
KU-5954	12894	3042	23804	15013	15765	5635	21600		25943	18517	14814	21417	89384	SNP
	359	67	736	455	468	149	708		781	537	462	716	3627	indel
KU-12180	21088	5156	38238	23831	25404	8469	36799	23377		20782	26378	29137	97676	SNP
	595	133	1140	753	752	264	1152	634		591	776	931	4144	indel
KU-12198	18187	4554	34198	21089	22352	8010	33356	18897	23133		22904	26792	98863	SNP
	511	99	1070	641	699	214	1057	500	677		653	851	3987	indel
KU-8-5	10895	2835	19706	13041	14112	4959	20817	12497	25989	19500		21747	89422	SNP
	302	80	656	456	477	136	739	359	784	577		693	3555	indel
KU-8-7	15466	4321	28539	18280	18723	6394	27463	16262	26137	20759	18595		92707	SNP
	450	114	878	577	545	198	875	469	771	607	558		3638	indel

Table S3 The number of SNPs and indels anchoerd to the barley chromosomes out of the SNPs and indels detected in each transcript-read pairing of 12 *Ae. umbellulata* accessions

Transcript model	Read													
	KU-4017	KU-4026	KU-4035	KU-4043	KU-4052	KU-4103	KU-5934	KU-5954	KU-12180	KU-12198	KU-8-5	KU-8-7	Total NR SNPs and indels	
KU-4017		2070<br>57	11395<br>346	7405<br>225	8243<br>234	3724<br>126	16938<br>562	9534<br>250	20699<br>563	15934<br>411	8817<br>264	17458<br>539	81098<br>3010	SNP<br>indel
KU-4026	7961<br>256		11843<br>453	8200<br>310	9530<br>314	4002<br>119	14074<br>558	8574<br>283	17356<br>546	12571<br>414	8145<br>302	14779<br>553	59840<br>2456	SNP<br>indel
KU-4035	6252<br>184	2471<br>62		7683<br>258	8960<br>278	3603<br>106	16769<br>574	10016<br>260	20768<br>563	15973<br>418	8437<br>247	17152<br>549	84827<br>3090	SNP<br>indel
KU-4043	6631<br>182	1931<br>49	11804<br>384		8361<br>285	3905<br>113	17296<br>561	10334<br>276	20877<br>574	15971<br>446	9044<br>294	17933<br>534	80931<br>3130	SNP<br>indel
KU-4052	7030<br>226	2405<br>62	13941<br>443	7620<br>243		3531<br>101	17500<br>536	10012<br>271	20884<br>616	16412<br>463	9997<br>304	17809<br>546	84172<br>3288	SNP<br>indel
KU-4103	8325<br>239	2837<br>76	13648<br>497	9885<br>349	9787<br>317		15754<br>580	9611<br>301	18809<br>549	14298<br>423	10062<br>338	15951<br>534	67222<br>2740	SNP<br>indel
KU-5934	9806<br>301	2333<br>58	17167<br>576	11198<br>368	11691<br>355	4324<br>124		9178<br>251	21342<br>586	15961<br>397	10610<br>345	17032<br>535	84658<br>3249	SNP<br>indel
KU-5954	10680<br>275	2563<br>68	19136<br>578	12220<br>366	13081<br>382	4588<br>114	17593<br>553		21075<br>599	15269<br>402	12109<br>343	17609<br>577	86210<br>3452	SNP<br>indel
KU-12180	17186<br>482	4121<br>104	30684<br>882	19075<br>573	20421<br>593	6841<br>210	29465<br>879	18909<br>497		16900<br>455	21166<br>608	23752<br>742	94043<br>3920	SNP<br>indel
KU-12198	14799<br>402	3482<br>77	27591<br>848	16760<br>498	17710<br>544	6297<br>173	26401<br>812	15484<br>379	18309<br>527		18265<br>514	21822<br>669	95281<br>3786	SNP<br>indel
KU-8-5	8838<br>255	2340<br>64	15502<br>534	10514<br>375	11544<br>390	4044<br>117	16887<br>595	10206<br>301	21099<br>618	15850<br>449		17202<br>554	85701<br>3396	SNP<br>indel
KU-8-7	12855<br>371	3621<br>95	23031<br>699	14675<br>481	15284<br>439	5251<br>156	21984<br>708	13533<br>408	21379<br>607	17168<br>461	14950<br>436		88909<br>3469	SNP<br>indel

,

Table S4 The number of non-redundant SNPs anchored to each *Ae. tauschii* chromosome

**Chr. number**	**Chr. length (Mbp)**	**number of SNPs**												
		**KU-4017**	**KU-4026**	**KU-4035**	**KU-4043**	**KU-4052**	**KU-4103**	**KU-5934**	**KU-5954**	**KU-12180**	**KU-12198**	**KU-8-5**	**KU-8-7**	**Ave.**
1	502.3	9775	7859	10119	9986	10599	8383	10368	10150	11832	12178	10368	11088	10225
2	651.7	12886	9181	13312	12391	13009	10234	12692	12623	14509	14882	13105	13320	12679
3	627.2	11078	8468	10956	10891	11520	9700	11137	11894	12818	12602	11553	11984	11217
4	526.0	6591	5256	6430	6648	6487	5725	6662	6759	7695	7643	6951	7237	6674
5	577.4	12159	9407	12345	12407	12511	10302	12751	13246	13627	14432	12663	13730	12465
6	496.0	9042	6667	9261	8905	8985	7636	9293	9413	10241	9964	9189	9495	9008
7	644.7	10608	8258	10711	10476	11323	9411	10693	11319	12674	11906	10766	11546	10808
Average	575.0	10306	7871	10448	10243	10633	8770	10514	10772	11914	11944	10656	11200	10439
total		72139	55096	73134	71704	74434	61391	73596	75404	83396	83607	74595	78400	73075
(%)		85.52	88.95	83.30	85.39	85.33	88.40	83.58	84.36	85.38	84.57	83.42	84.57	85.07
ummapped		12210	6846	14661	12269	12796	8058	14455	13980	14280	15256	14827	14307	12829
(%)		14.48	11.05	16.70	14.61	14.67	11.60	16.42	15.64	14.62	15.43	16.58	15.43	14.93

Table S5 The number of non-redundant SNPs anchored to each barley chromosome

**Chr. number**	**Chr. length (Mbp)**	**number of SNPs**												
		**KU-4017**	**KU-4026**	**KU-4035**	**KU-4043**	**KU-4052**	**KU-4103**	**KU-5934**	**KU-5954**	**KU-12180**	**KU-12198**	**KU-8-5**	**KU-8-7**	**Ave.**
1	464.1	7941	6292	7580	8002	8189	6620	8193	8150	9031	8889	7875	8521	7940
2	623.8	9648	6964	10173	9310	9410	7976	9518	9709	10819	11251	9781	9605	9514
3	558.7	9201	7177	9303	9333	9663	8149	9584	9801	10917	10483	9679	10332	9469
4	543.8	5458	4525	5219	5290	5481	4970	5397	5617	6502	6379	5654	5940	5536
5	558.6	9901	7582	9756	9733	10161	8359	9858	10896	11164	11364	10010	10452	9936
6	538.5	6801	5369	7107	6846	7071	6206	7582	7546	7923	7566	7079	7734	7069
7	599.7	8599	7039	8606	8832	9145	7651	8824	8746	9929	9734	8905	9387	8783
Average	555.3	8221	6421	8249	8192	8446	7133	8422	8638	9469	9381	8426	8853	8321
total		57549	44948	57744	57346	59120	49931	58956	60465	66285	65666	58983	61971	58247
(%)		70.96	75.11	68.07	70.86	70.24	74.28	69.64	70.14	70.48	68.92	68.82	69.70	70.40
ummapped		23549	14892	27083	23585	25052	17291	25702	25745	27758	29615	26718	26938	24494
(%)		29.04	24.89	31.93	29.14	29.76	25.72	30.36	29.86	29.52	31.08	31.18	30.30	29.60

Table S6 Summary of nucleotide polymorphism and divergence in *Ae. umbellulata*, *Ae. tauschii* and *T. urartu*

Reference accession	*Ae. umbellulata* KU-4017			*Ae. tauschii* KU-2075		
Species to be compared (1 vs. 2)	*Ae. umbellulata* vs *Ae. tauschii*	*Ae. umbellulata* vs *T. urartu*	*T. urartu* vs *Ae. tauschii*	*Ae. umbellulata* vs *Ae. tauschii*	*Ae. umbellulata* vs *T. urartu*	*T. urartu* vs *Ae. tauschii*
# of fixed differences	12767	17127	17133	12312	16233	16136
Polymorphic sites in *Ae. umbellulata* ($n^b = 12$)	4500	4762	NA[a]	4103	4317	NA
Polymorphic sites in *Ae. tauschii* ($n^b = 10$)	3891	NA	4153	3622	NA	3836
Shared polymorphic sites	262	NA	NA	214	NA	NA
Average number of nucleotide differences between populations	16201.033	19043.583	18805.1	15463.208	17932.25	17673.3

[a]Not applied; [b]Number of accessions.

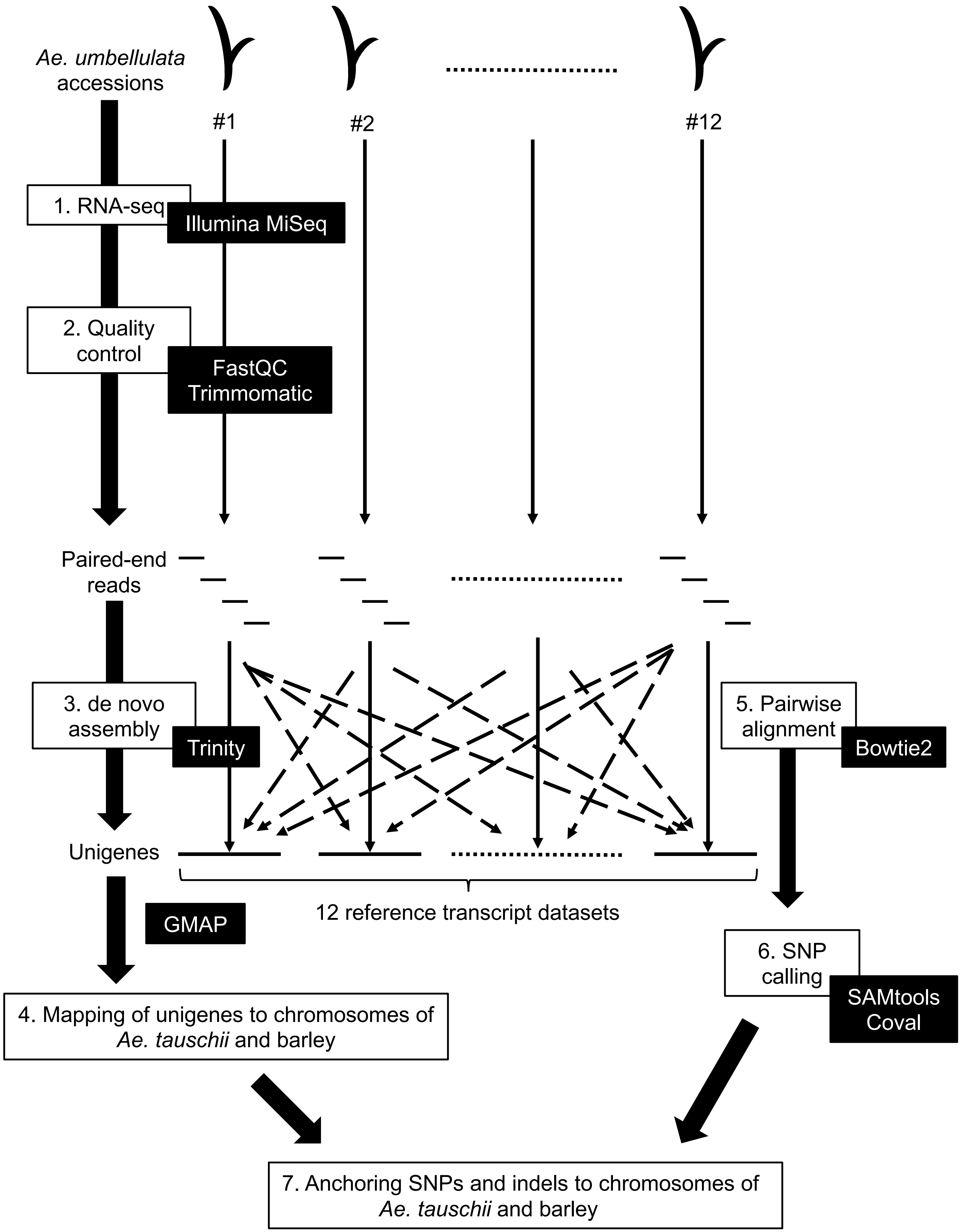

Fig. S1 The workflow of RNA-seq analysis

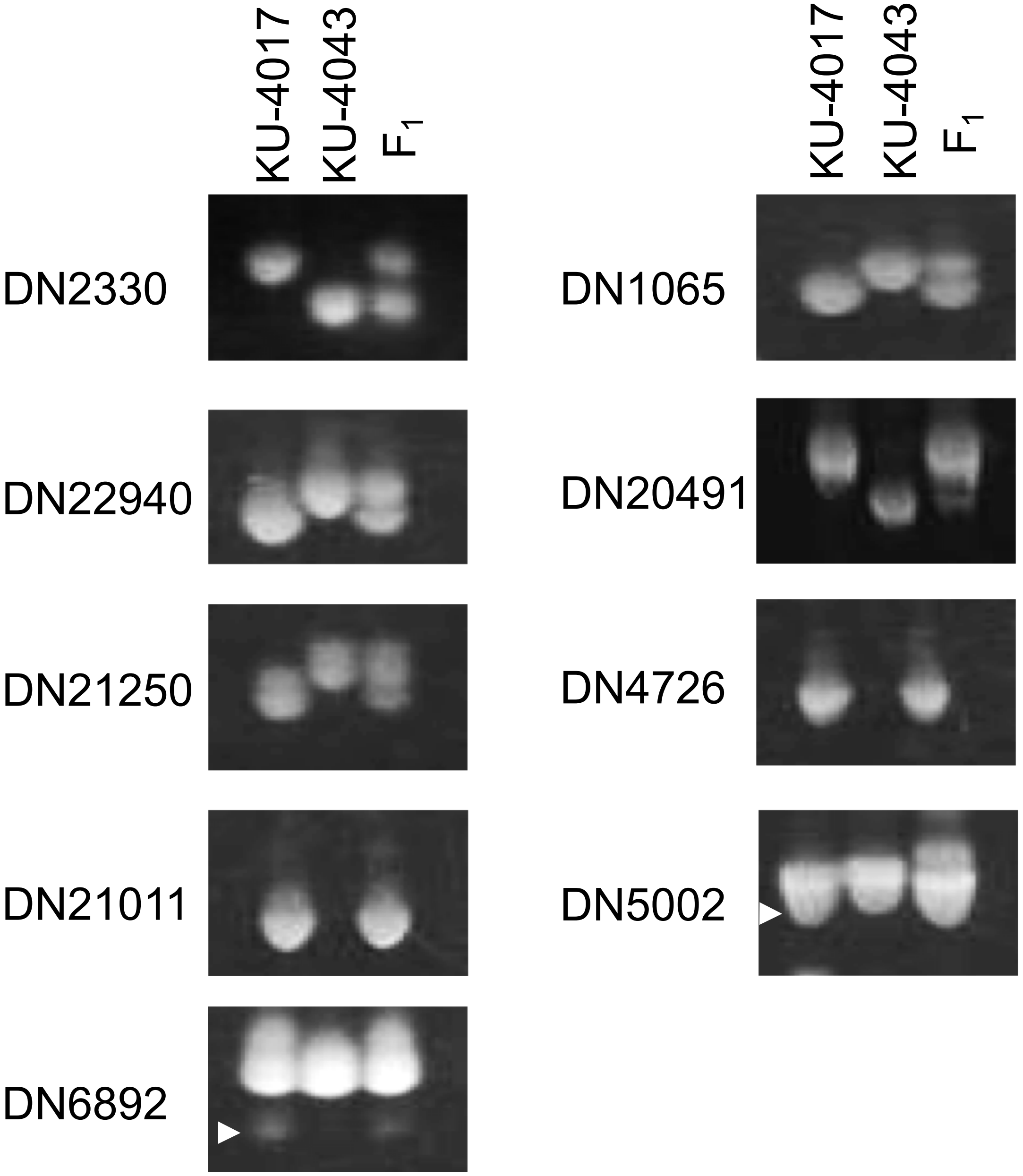

Fig. S2 Images of polyacrylamide gel electrophoresis for indel markers. PCR amplification products of the F1 plant and its parent accession of KU-4017 and KU-4043 for each marker were loaded in 20% polyacrylamide gel. The marker names are shown on the left of each gel image. The size difference between the parents was observed in the markers DN2330, DN1065. DN22940, DN20941, and DN21250. Presence/absence of amplicons between the parents was detected in the markers DN4726 and DN21011. An extra band of KU-4017 appeared in the marker DN5002 and DN6892 (shown by white arrowheads).

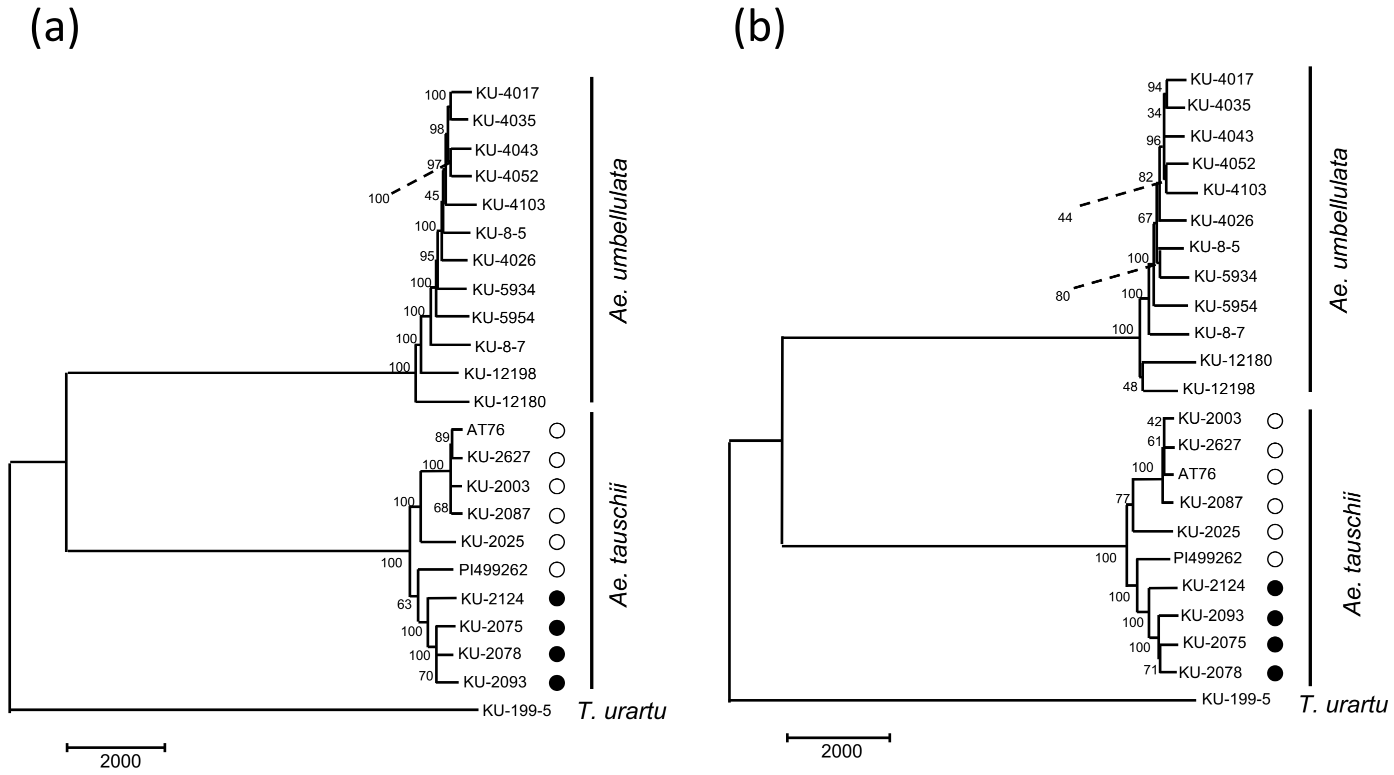

Fig. S3 Phylogenetic relationship between 12 *Ae. umbellulata* accessions, 10 *Ae. tauschii* accessions and one *T. urartu* accession based on SNPs that was estimated by using the *Ae. umbellulata* KU-4017 reference transcript dataset (**a**) and the *Ae. tauschii* KU-2075 reference transcript dataset (**b**). These trees were constructed by Neighbor-Joining method. The bootstrap value (1000 replicates) are shown on the branch. White and black circles of *Ae. tauschii* are corresponding to the divergent lineages TauL1 and TauL2 (Matsuoka et al. 2015), respectively. *T. urartu* was used as the outgroup species.

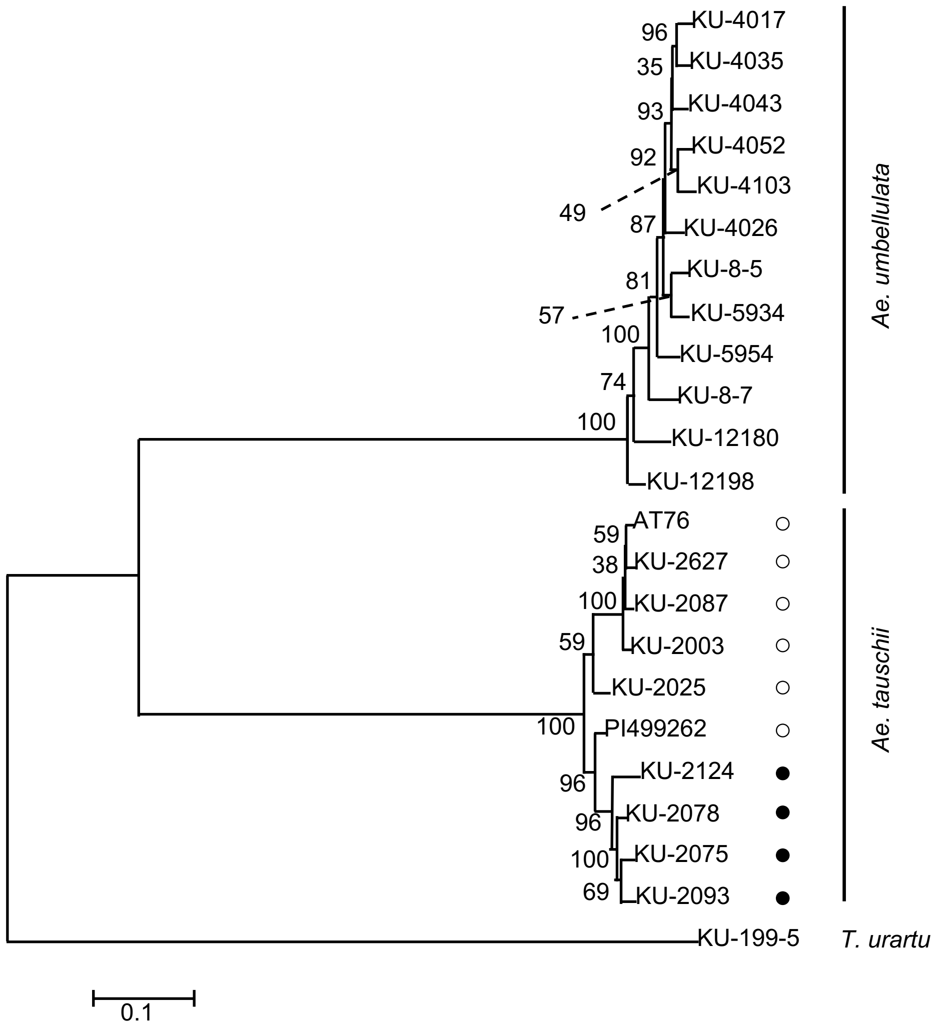

Fig. S4 Phylogenetic relationship between the 12 *Ae. umbellulata* accessions, 10 *Ae. tauschii* accessions and one *T. urartu* accession based on SNPs estimated using the *Ae. tauschii* KU-2075 reference transcript dataset. The tree was constructed by the maximum-likelihood method. The bootstrap values (1000 replicates) are shown on each branch. White and black circles of *Ae. tauschii* respectively correspond to the divergent lineages TauL1 and TauL2 [39]. *T. urartu* was used as the outgroup species.

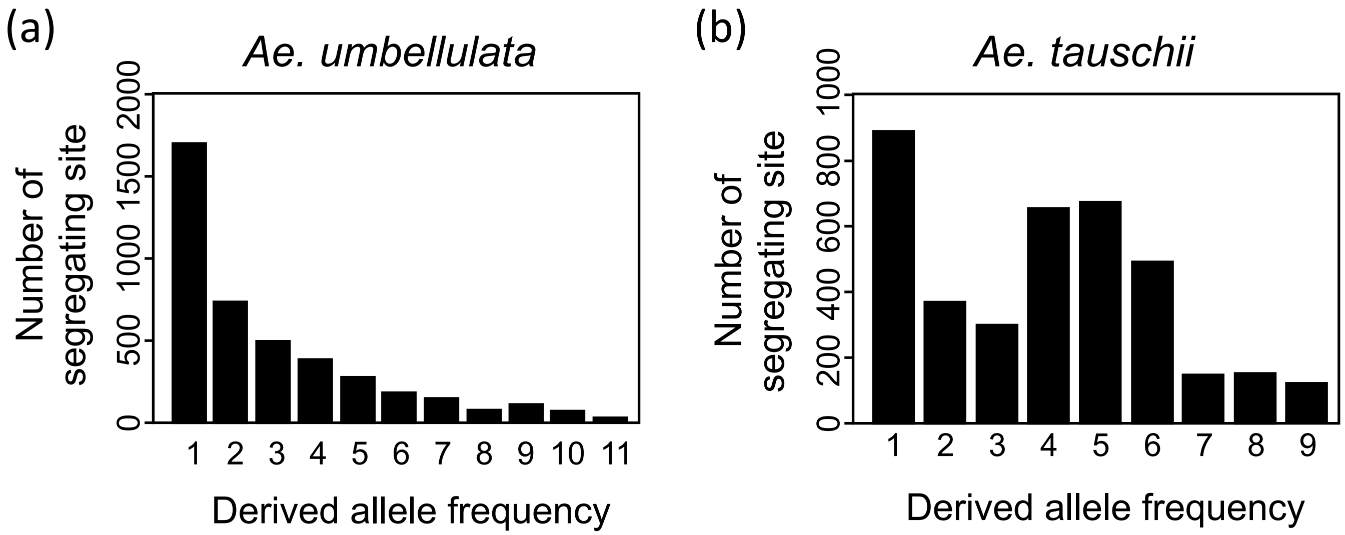

Fig. S5 Derived allele frequency distribution in *Ae. umbellulata* (n = 12) (a) and *Ae. tauschii* (n = 10) (b), respectively. *Ae. tauschii* KU-2075 transcripts were used as the reference. Derived alleles were estimated using the outgroup species *T. urartu*.
